# Modeling Group Size and Scalar Stress by Logistic Regression from an Archaeological Perspective

**DOI:** 10.1371/journal.pone.0091510

**Published:** 2014-03-13

**Authors:** Gianmarco Alberti

**Affiliations:** Independent Researcher, Catania, Italy; Universidad Carlos III de Madrid, Spain

## Abstract

Johnson’s scalar stress theory, describing the mechanics of (and the remedies to) the increase in in-group conflictuality that parallels the increase in groups’ size, provides scholars with a useful theoretical framework for the understanding of different aspects of the material culture of past communities (i.e., social organization, communal food consumption, ceramic style, architecture and settlement layout). Due to its relevance in archaeology and anthropology, the article aims at proposing a predictive model of critical level of scalar stress on the basis of community size. Drawing upon Johnson’s theory and on Dunbar’s findings on the cognitive constrains to human group size, a model is built by means of Logistic Regression on the basis of the data on colony fissioning among the Hutterites of North America. On the grounds of the theoretical framework sketched in the first part of the article, the absence or presence of colony fissioning is considered expression of not critical vs. critical level of scalar stress for the sake of the model building. The model, which is also tested against a sample of archaeological and ethnographic cases: a) confirms the existence of a significant relationship between critical scalar stress and group size, setting the issue on firmer statistical grounds; b) allows calculating the intercept and slope of the logistic regression model, which can be used in any time to estimate the probability that a community experienced a critical level of scalar stress; c) allows locating a critical scalar stress threshold at community size 127 (95% CI: 122–132), while the maximum probability of critical scale stress is predicted at size 158 (95% CI: 147–170). The model ultimately provides grounds to assess, for the sake of any further archaeological/anthropological interpretation, the probability that a group reached a hot spot of size development critical for its internal cohesion.

## Introduction

In anthropological and archaeological literature considerable attention is paid to the relationship between human groups size and different aspects of past material culture and social organization. Unlike previous works focusing on the connection between size and complexity (overview in [1], [2]), R. Rappaport and G. Johnson firstly and explicitly stressed the existence of limits to groups size due to communication strains, as Bandy notes [3]. On the grounds of different ethnographic case studies, they termed by *irritation coefficient* [4] and *scalar stress* [5], [6] the increase in in-group conflictuality that parallels the increase in groups’ size. Johnson extensively elaborated on the issue and on its numerical aspects, and framed the phenomenon in terms of groups’ decision-making. Simply put, given that daily interactions in human groups are based on communications between individuals, and that communication can be conceived as an information flow, a group arrives at a consensual decision by means of a face-to-face flow of information. This geometrically increases as the number of individuals increases, becoming unmanageable beyond a certain threshold. Further, to Johnson, human groups may address scale-related issues by either fission or group reorganization, which will be reviewed later in this work.

From an anthropological standpoint, a number of scholars have further underscored the existence of a group-size threshold, supporting Johnson’s notion of scalar stress. In his study of early village societies in the Bolivia’s Titicaca Basin, Bandy [3] notes that among the Siuai (New Guinea) village fissioning depends on the frequency of quarrelling, while Fry [7], [8] underscores that conflicts, though managed without violence, are widespread among small-size nomadic groups. Holmberg [9], for example, notes that among the Siriono, hunter-gatherers of eastern Bolivia, in-group conflicts occur between all types of people, relatives and nonrelatives. Groups may fission when tensions between individuals become intense. Among the Yanomama, South American Indians, Chagnon [10] notes that intravillage conflicts arise when the number of inhabitants rises above 200. By the same token, Bowser [11] stresses that in the Ecuadorian community of Conambo (about 200 people living in 25 households) conflicts often break up for different causes such as, e.g., marriage requests, fight between young men for jealousy, rights of new families to move into the community.

**Figure 1:**
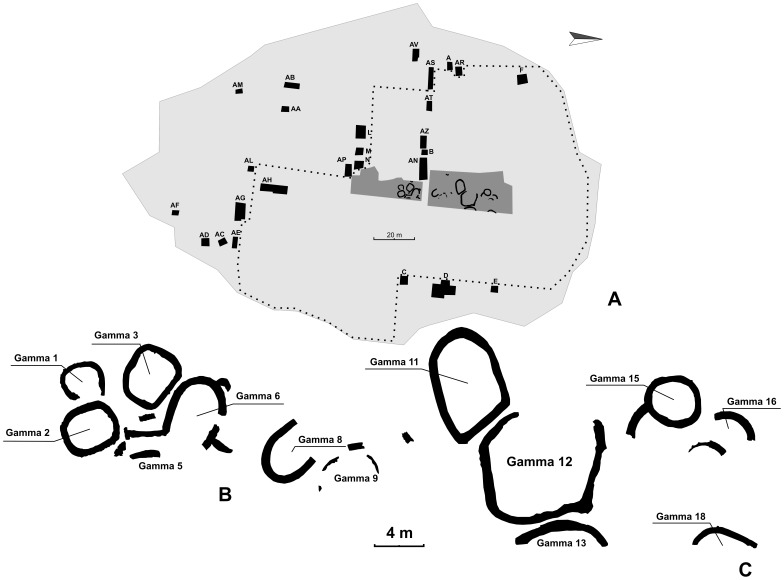
Example of integrative facility at the Middle Bronze Age settlement at Lipari (north-eastern Sicily, Italy). **A**) Main excavation areas (dark grey), trenches (black), limits of MBA occupation (dotted line). **B–C**) Layout of the southern (**B**) and northern (**C**) sector of the settlement. In evidence (larger label) the oversized polygonal structure (Gamma 12) possibly used as integrative facility according to Alberti’s analysis [38] (A–C drawing by the Author after [101]).

From an archaeological perspective, even though scalar stress theory has been criticized (e.g., [12]) for overlooking the contribution of agency [13], [14] in the process of social organization, and acknowledging the fact that indicators of scalar stress can be difficult to identify archaeologically [15], Johnson’s findings continue to provide scholars with a useful theoretical framework for the understanding of many aspects of the life and material culture of past communities, like social organization [16]–[19], stylistic display [15], [20]–[22], communal food consumption [23]–[25], architecture and settlement layout [3], [16], [20], [24], [26]–[33]. As Ames [17] notes, scalar stress is considered one of the proximate causes of the origin and development of social inequality and complexity, since it allows leadership to emerge to ameliorate scale-related social problems (see also [16], [18]). Hegmon [20], [21] locates a connection between scalar stress and ceramic style, arguing that the latter, as vehicle of social identity, may ameliorate scale-related issues by promoting the communication flow between socially distant individuals. By the same token, Nelson et al. [22] underscore the relevance of style in consensual decision making for its ability to promote a sense of sameness among interacting individuals and to enhance group cohesion. As for feasting, Lee [24], for instance, underscores the connection between large structures at the Neolithic settlement of Jiangzhai (China) and material remains related to shared food consumption, considering both as critical for the community’s cohesion. As far as architecture and settlement layout is concerned, Adler and Wilshusen [30], [34] have located a connection between scale-related social issues and the use of structures for the integration of individuals above the household level, which they termed integrative facilities. These are places where scalar stress-reduction practices are put to work in the context of information control and decision-making. As the scholars underscore, expanding a Johnson’s idea [5] and building upon Rappaport [35] and Turner’s [36] view of ritual, inasmuch rituals can be conceived as sequences of redundant and invariant acts, they can ameliorate scalar stress by promoting an effective communication flow and by fostering in-group consensus and cohesion. Drawing upon Adler and Wilshusen’s findings, integrative facilities aimed at counteracting divisive social forces, integrating people at different levels, and promoting social bonds, have been identified by scholars working in different cultural and chronological horizons (see also [37] for an overview), like Sicily [38], Neolithic Greece [39], Anatolia [40], Near East [32], [41]–[43], China [24], Mongolian Steppe [27], pre-contact North [26], [28], [29], [37], [44] and South America [3], [31] (Fig. 1).

### Aim of the Study

Given the importance of Johnson’s theory of scalar stress for the understanding of many aspects of past communities’ organization and material culture, this work aims at building a predictive model [45], [46] of scalar stress that can be put to work when estimates of settlement population are available. A predictive model can prove useful when one wishes to predict the probability that a settlement experienced scale-related issues, i.e. it was past the aforementioned communication-strain threshold, therefore experiencing critical scalar stress. A predictive model would so provide a framework for a better understanding and interpretation of evidences like integrative facilities or other material remains of possible integrative nature. Acknowledging that fact that the estimation of a site’s population on archaeological grounds is a thorny problem (e.g., [47], [48]–[52]) and that this could condition the application of the model to archaeological case, as a matter of fact archaeologists are often in the position to arrive at an estimate of the number of residents (e.g., [3], [24], [26], [40], [53], [54]–[56]). This makes feasible the use of the proposed model. If population size can be estimated beforehand, e.g. by probabilistic sampling [53], survey data [3], projection of the exposed residential area [24], [55], [56] or other approaches (e.g., [57], [58]), then the model can provide the basis to predict the probability of experiencing critical scalar stress, allowing expecting the presence of evidence of integrative mechanisms (e.g., integrative facilities) and hence possibly tailoring the researches on field. If evidences pointing to mechanisms of possible integrative nature are documented, the predicted probability returned by the model can provide grounds for evaluating if a critical level of communication stress was reached and could therefore account for such evidences.

It must be stressed at the outset that while the practices put to work by groups to counteract stress represent interesting fields of inquiry, they are beyond the scope of this study, which rather focuses on building a predictive model of scalar stress. Although at a descriptive level, many types of human responses to scale-related issues are reported both earlier and later on in this article, such as fission, hierarchization, development of integrative mechanisms, stylistic display, and communal consumption, none of which I intend to directly address.

The goal of the study is achieved by the following steps, which make up the remainder of this article. First, I review the Johnson’s theory of scalar stress, highlighting its cognitive bases and numerical aspects. Also, I put his theory in relation with the Dunbar’s model of cognitive limits to group size [59]; this is done for two reasons: (a) because this model is germane to the Johnson’s scalar stress perspective (e.g., [1]), and (b) because Dunbar’s model allows translating (so to say) Johnson’s theory in terms of sheer number of individuals rather than decision-making units. After, a predictive model for scalar stress is built by means of logistic regression [60]–[62] on the basis of the data derived from the Olsen’s study [63] of the cycles of community fission among the Hutterites of North America [64]–[66], which can be considered [59], [67]–[69] evidence of the constrains put to group size by human cognitive limits. The model is then discussed and tested against both archaeological and ethnographical data.

### Theoretical Framework

To Johnson [5], there seems to exist a threshold in groups’ size above which communication flow becomes unmanageable. In locating around 6 that threshold, he relied upon previous studies [70]–[72] in cognitive psychology and small-group dynamics (overview in [73], [74], [75]). These have pointed out that during decision-making, the quality of task solution increases with group size because larger groups have higher probability that someone will have pieces of information essential to the problem’s solution [76]. Nonetheless, as group’s size increases, the quality of the decision drops fast [72], [77]. Members see larger groups as too large for an effective task performance, having too much competition, disunity, disagreement [70], [71] and communicative difficulties that create stress on individuals [78]. As size increases, groups tend to form sub-groups, which lead to a drop of overall cohesion [70], [79] and cooperative consensus since larger groups are more likely to contain noncooperative individuals [80].

In Johnson’s view, one option to mitigate scalar stress is by fissioning into smaller groups in order to reduce the number of decision-making units. He considered this option a common response to scalar stress, unless intervening factors do not discourage group fission. In this respect, Bowser [11] and Bandy [3], for instance, have underscored that fission has downsides in terms of economic and social costs, and that factors like high levels of external conflicts, investment in nonportable capital, and high population density constrain fissioning. On the grounds of the ethnographic data, Johnson [5], [6] argued that a way to reduce scalar stress without fissioning is to reorganize decision-making structure into sequential hierarchies, that is by grouping individuals into a smaller number of more inclusive decision-making units in such a way that their number would still centred round 6. In this scheme, the decision-making flow would move bottom up, involving consensual decisions at each step. Remarkably, Reynolds [81] has formally showed that nested levels of decision can speed up problems’ solution by their ability to partition the problem into sub-problems that can further solved each at every different organizational level, so supporting Johnson’s findings.

It has to be noted that scale-related issues do not mechanically dictate the types and forms of human behaviour, since Johnson’s decision-making reshaping can be thought of as occurring under specific conditions. It could be considered the middle stage between fission and the emergence of non-consensual (i.e., hierarchical) decision-making bodies. As stressed by Lee Lyman [18], group fissioning could be the immediate choice for aggregates experiencing scale-related issue but, when the landscape fills in or mobility is otherwise limited, sequential hierarchies would then evolve, eventually followed by a more vertical decision-making organization when the sequential one proves unable to further reduce scalar stress. In this respect, in fact, the number of sequential decision-making units cannot grow larger indefinitely since consensus must be reached at a greater number of operational levels, as noted by Johnson [5]. Other factors may shape the response to scale-related issues, as the ones highlighted by Friesen [16] who argues that the development of sequential hierarchies as remedy to scalar stress can be favoured by a lack of economic and resource conditions that could allow formal leaders to emerge.

Evolutionary psychologist R. Dunbar [59], [68], [69] has also located cognitive limits to groups’ size from a *social channel capacity* [67] perspective or, in other words, from the standpoint of the limits of human brain ability to handle social networks of increasing size. This turns out to be germane, in my opinion, to the Johnson’s view of face-to-face information flow in decision-making context. While the first model refers to the number of decision-makers, the second can be conceptualized as working at the level of absolute population size. This link will become apparent later on, when I will describe the Hutterite data on which the proposed model is based. Dunbar’s findings predict that the average size of human groups, where cohesion is maintained without complicated rules and regulations, should be centred around 150, with 100 and 230 as minimum and maximum figures. He has found that this threshold recursively occurs in examples of human aggregates from archaeology and history as, for instance, among the Hutterites, an Anabaptist group settled in North America in the 1800s and organized into agricultural colonies grouped into three endogamous subsets (Lehrerleut, Schmiedenleut and Dariulsleut). Hutterite colonies usually fission at a population threshold that Olsen [63] locates somewhere between 150 and 175 persons. In this respect, Gladwell [67] interestingly reports the opinion of a leader of one of such colonies who noted that when size increases *people become strangers to one another*, whereas *in smaller groups people are a lot closer.* He notes that *they’re knit together, which is very important if you want to be effective and successful at community life*, and goes on saying that if a colony grows too large *you don’t have enough things in common, and then you start to become strangers and that close-knit fellowship starts to get lost*.

While it is true that *many human groups exceed* the Dunbar’s threshold [82], it is worth noting that his model does leave room for that possibility, provided that groups find a way to counteract the divisive forces that increase with increasing group size [83], as Johnson has pointed out. In this respect, Carneiro [84] (see also [5]) contrasts the case of the Yanomama villages (fissioning when size rises above 200) to that of the Kayapò (whose villages reach 600/800 inhabitants), arguing that what accounts for the difference is the organization of the second into nested social segments. The latter, in line with Johnson’s model of sequential hierarchies, allow reducing the load of information processed by each segment, relieving *the social system of the need to process loads of information that would exceed individuals’ cognitive capacities*, as Dubreuil puts it [85].

Dunbar’s findings are compatible with both earlier and later studies pointing to the existence of limits to the number of subjects that can be integrated in a relational network [86]–[88]. It is worthy of note that, from an anthropological and archaeological perspective, Kosse [89], [90] has also argued that, due to limits of long-term memory, a first critical threshold for the load of information flow in one-to-one relations should occur at group size somewhere between 100 and 200, and that above that size groups are expected to develop integrative mechanisms to maintain cohesion. As put by Dunbar [68], what constrains group size is not just a matter of memory, but the limited ability to manipulate increasing amount of information. In fact, as Gladwell [67] stresses, being a group means that *you have to understand the personal dynamics of the group, juggle different personalities, keep people happy, manage demands on your time and attention*; as consequence, *even a relatively small increase in the size of a group […] creates a significant additional social and intellectual burden*.

## Materials and Methods

For the purposes of this study, logistic regression is used to build a predictive model for scalar stress on the basis of population size. To put it in a nutshell, and referring the readers to the literature previously quoted for an in-deep treatment of the topic, logistic regression is a statistical technique that finds use also in archaeology (e.g., [91], [92]) and allows estimating the probability that a particular outcome of a dependent nominal variable *y* will occur based on information from one (or more) explanatory variable *x*. It is analogous to linear regression, except that the dependent variable is nominal, not a measurement. The technique ultimately finds the equation that best predicts the probability *p* of getting a particular value of *y*, with *p* taking values from 0 to 1. The general form of the logistic regression model is:
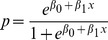



Unlike the least-squares method used in linear regression, logistic regression finds the intercept (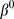) and slope (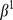) of the best-fitting equation by means of the maximum-likelihood method, which is a computer-intensive technique that finds *the values of the parameters under which you would be most likely to get the observed results* [61] (see also [93]). Once logistic regression has been ran, and the intercept and slope have been found, one is in the position to derive the probability of the outcome of *y* by plugging those two parameters and any known value(s) of *x* into the logistic regression model.

To determine the degree to which the model fits, the following steps must be taken [60], [94]: a) verify the results of the chi-square goodness-of-fit test, and conclude that the model fits if the associated *p* value is greater than 0.05 (i.e., there is no significant difference between what the model predicts and what the analyst observes in the data); b) check the significance of the intercept and slope; c) check the association between concordant pairs, i.e. *the proportion of times the data and the model actually agree with each other* [94]. Additionally, a classification table (reporting the overall percentage of correctly classified cases) is usually also reported in order to assess the classificatory power of the model (e.g., [92]).

As previously noted, the model here proposed has been built drawing upon the data derived from the study by Olsen [63], which reports the size of the Lehrerleut and Schmiedenleut colonies both at and after fissioning (Table 1).

**Table 1:** Data about the size of the Hutterites’ colonies at and after fissioning (derived from Olsen [63]), used to build the Logistic Regression model object of this article.

n of indiv	at/after fissioning	endog. subset	n of indiv	at/after fissioning	endog. subset
196	at fissioning	Lehrerleut	92	after fissioning	Lehrerleut
197	at fissioning	Lehrerleut	88	after fissioning	Lehrerleut
180	at fissioning	Lehrerleut	103	after fissioning	Lehrerleut
170	at fissioning	Lehrerleut	85	after fissioning	Lehrerleut
188	at fissioning	Lehrerleut	78	after fissioning	Lehrerleut
176	at fissioning	Lehrerleut	72	after fissioning	Lehrerleut
161	at fissioning	Lehrerleut	86	after fissioning	Lehrerleut
165	at fissioning	Lehrerleut	86	after fissioning	Lehrerleut
150	at fissioning	Lehrerleut	92	after fissioning	Lehrerleut
131	at fissioning	Lehrerleut	94	after fissioning	Lehrerleut
152	at fissioning	Lehrerleut	96	after fissioning	Lehrerleut
136	at fissioning	Lehrerleut	75	after fissioning	Lehrerleut
179	at fissioning	Lehrerleut	99	after fissioning	Lehrerleut
182	at fissioning	Lehrerleut	91	after fissioning	Lehrerleut
186	at fissioning	Lehrerleut	69	after fissioning	Lehrerleut
187	at fissioning	Lehrerleut	133	after fissioning	Lehrerleut
187	at fissioning	Lehrerleut	105	after fissioning	Lehrerleut
171	at fissioning	Lehrerleut	84	after fissioning	Lehrerleut
155	at fissioning	Lehrerleut	72	after fissioning	Lehrerleut
174	at fissioning	Lehrerleut	70	after fissioning	Lehrerleut
190	at fissioning	Lehrerleut	79	after fissioning	Lehrerleut
147	at fissioning	Lehrerleut	45	after fissioning	Lehrerleut
173	at fissioning	Lehrerleut	79	after fissioning	Lehrerleut
202	at fissioning	Lehrerleut	78	after fissioning	Lehrerleut
179	at fissioning	Lehrerleut	71	after fissioning	Lehrerleut
142	at fissioning	Lehrerleut	66	after fissioning	Lehrerleut
136	at fissioning	Lehrerleut	80	after fissioning	Lehrerleut
163	at fissioning	Lehrerleut	84	after fissioning	Lehrerleut
190	at fissioning	Lehrerleut	77	after fissioning	Lehrerleut
185	at fissioning	Lehrerleut	88	after fissioning	Lehrerleut
189	at fissioning	Lehrerleut	72	after fissioning	Lehrerleut
140	at fissioning	Lehrerleut	78	after fissioning	Lehrerleut
157	at fissioning	Lehrerleut	80	after fissioning	Lehrerleut
142	at fissioning	Lehrerleut	90	after fissioning	Lehrerleut
154	at fissioning	Lehrerleut	70	after fissioning	Lehrerleut
155	at fissioning	Lehrerleut	66	after fissioning	Lehrerleut
124	at fissioning	Lehrerleut	89	after fissioning	Lehrerleut
133	at fissioning	Lehrerleut	87	after fissioning	Lehrerleut
157	at fissioning	Lehrerleut	86	after fissioning	Lehrerleut
153	at fissioning	Lehrerleut	75	after fissioning	Lehrerleut
209	at fissioning	Lehrerleut	63	after fissioning	Lehrerleut
137	at fissioning	Lehrerleut	68	after fissioning	Lehrerleut
154	at fissioning	Lehrerleut	68	after fissioning	Lehrerleut
164	at fissioning	Lehrerleut	68	after fissioning	Lehrerleut
207	at fissioning	Lehrerleut	94	after fissioning	Lehrerleut
150	at fissioning	Lehrerleut	93	after fissioning	Lehrerleut
165	at fissioning	Lehrerleut	93	after fissioning	Lehrerleut
135	after fissioning	Lehrerleut	94	after fissioning	Lehrerleut
127	after fissioning	Lehrerleut	79	after fissioning	Lehrerleut
125	after fissioning	Lehrerleut	76	after fissioning	Lehrerleut
84	after fissioning	Lehrerleut	69	after fissioning	Lehrerleut
94	after fissioning	Lehrerleut	78	after fissioning	Lehrerleut
102	after fissioning	Lehrerleut	82	after fissioning	Lehrerleut
108	after fissioning	Lehrerleut	91	after fissioning	Lehrerleut
71	after fissioning	Lehrerleut	74	after fissioning	Lehrerleut
111	after fissioning	Lehrerleut	68	after fissioning	Lehrerleut
79	after fissioning	Lehrerleut	75	after fissioning	Lehrerleut
79	after fissioning	Lehrerleut	61	after fissioning	Lehrerleut
74	after fissioning	Lehrerleut	82	after fissioning	Lehrerleut
102	after fissioning	Lehrerleut	81	after fissioning	Lehrerleut
95	after fissioning	Lehrerleut	87	after fissioning	Lehrerleut
92	after fissioning	Lehrerleut	92	after fissioning	Lehrerleut
73	after fissioning	Lehrerleut	70	after fissioning	Lehrerleut
99	after fissioning	Lehrerleut	70	after fissioning	Lehrerleut
82	after fissioning	Lehrerleut	79	after fissioning	Lehrerleut
87	after fissioning	Lehrerleut	78	after fissioning	Lehrerleut
87	after fissioning	Lehrerleut	81	after fissioning	Lehrerleut
94	after fissioning	Lehrerleut	73	after fissioning	Lehrerleut
91	after fissioning	Lehrerleut	69	after fissioning	Lehrerleut
86	after fissioning	Lehrerleut	64	after fissioning	Lehrerleut
69	after fissioning	Lehrerleut	79	after fissioning	Lehrerleut
121	after fissioning	Lehrerleut	75	after fissioning	Lehrerleut
88	after fissioning	Lehrerleut	251	at fissioning	Schmiedenleut
99	after fissioning	Lehrerleut	208	at fissioning	Schmiedenleut
108	after fissioning	Lehrerleut	185	at fissioning	Schmiedenleut
212	at fissioning	Schmiedenleut	87	after fissioning	Schmiedenleut
197	at fissioning	Schmiedenleut	84	after fissioning	Schmiedenleut
128	at fissioning	Schmiedenleut	57	after fissioning	Schmiedenleut
143	at fissioning	Schmiedenleut	83	after fissioning	Schmiedenleut
177	at fissioning	Schmiedenleut	82	after fissioning	Schmiedenleut
130	at fissioning	Schmiedenleut	103	after fissioning	Schmiedenleut
169	at fissioning	Schmiedenleut	100	after fissioning	Schmiedenleut
202	at fissioning	Schmiedenleut	91	after fissioning	Schmiedenleut
155	at fissioning	Schmiedenleut	54	after fissioning	Schmiedenleut
169	at fissioning	Schmiedenleut	86	after fissioning	Schmiedenleut
183	at fissioning	Schmiedenleut	73	after fissioning	Schmiedenleut
204	at fissioning	Schmiedenleut	107	after fissioning	Schmiedenleut
181	at fissioning	Schmiedenleut	84	after fissioning	Schmiedenleut
172	at fissioning	Schmiedenleut	89	after fissioning	Schmiedenleut
164	at fissioning	Schmiedenleut	83	after fissioning	Schmiedenleut
141	at fissioning	Schmiedenleut	107	after fissioning	Schmiedenleut
133	at fissioning	Schmiedenleut	102	after fissioning	Schmiedenleut
188	at fissioning	Schmiedenleut	102	after fissioning	Schmiedenleut
165	at fissioning	Schmiedenleut	106	after fissioning	Schmiedenleut
158	at fissioning	Schmiedenleut	82	after fissioning	Schmiedenleut
172	at fissioning	Schmiedenleut	72	after fissioning	Schmiedenleut
174	at fissioning	Schmiedenleut	84	after fissioning	Schmiedenleut
141	at fissioning	Schmiedenleut	76	after fissioning	Schmiedenleut
203	at fissioning	Schmiedenleut	80	after fissioning	Schmiedenleut
187	at fissioning	Schmiedenleut	65	after fissioning	Schmiedenleut
136	at fissioning	Schmiedenleut	120	after fissioning	Schmiedenleut
151	at fissioning	Schmiedenleut	77	after fissioning	Schmiedenleut
110	at fissioning	Schmiedenleut	64	after fissioning	Schmiedenleut
142	at fissioning	Schmiedenleut	64	after fissioning	Schmiedenleut
145	at fissioning	Schmiedenleut	74	after fissioning	Schmiedenleut
104	at fissioning	Schmiedenleut	69	after fissioning	Schmiedenleut
159	at fissioning	Schmiedenleut	100	after fissioning	Schmiedenleut
144	at fissioning	Schmiedenleut	77	after fissioning	Schmiedenleut
162	at fissioning	Schmiedenleut	82	after fissioning	Schmiedenleut
191	at fissioning	Schmiedenleut	48	after fissioning	Schmiedenleut
162	at fissioning	Schmiedenleut	105	after fissioning	Schmiedenleut
145	at fissioning	Schmiedenleut	97	after fissioning	Schmiedenleut
180	at fissioning	Schmiedenleut	76	after fissioning	Schmiedenleut
188	at fissioning	Schmiedenleut	79	after fissioning	Schmiedenleut
172	at fissioning	Schmiedenleut	100	after fissioning	Schmiedenleut
209	at fissioning	Schmiedenleut	69	after fissioning	Schmiedenleut
208	at fissioning	Schmiedenleut	67	after fissioning	Schmiedenleut
161	at fissioning	Schmiedenleut	94	after fissioning	Schmiedenleut
133	at fissioning	Schmiedenleut	91	after fissioning	Schmiedenleut
160	at fissioning	Schmiedenleut	81	after fissioning	Schmiedenleut
145	at fissioning	Schmiedenleut	82	after fissioning	Schmiedenleut
108	after fissioning	Schmiedenleut	82	after fissioning	Schmiedenleut
48	after fissioning	Schmiedenleut	71	after fissioning	Schmiedenleut
164	after fissioning	Schmiedenleut	62	after fissioning	Schmiedenleut
87	after fissioning	Schmiedenleut	84	after fissioning	Schmiedenleut
86	after fissioning	Schmiedenleut	74	after fissioning	Schmiedenleut
65	after fissioning	Schmiedenleut	102	after fissioning	Schmiedenleut
118	after fissioning	Schmiedenleut	70	after fissioning	Schmiedenleut
90	after fissioning	Schmiedenleut	83	after fissioning	Schmiedenleut
74	after fissioning	Schmiedenleut	58	after fissioning	Schmiedenleut
36	after fissioning	Schmiedenleut	94	after fissioning	Schmiedenleut
106	after fissioning	Schmiedenleut	80	after fissioning	Schmiedenleut
74	after fissioning	Schmiedenleut	119	after fissioning	Schmiedenleut
127	after fissioning	Schmiedenleut	106	after fissioning	Schmiedenleut
58	after fissioning	Schmiedenleut	81	after fissioning	Schmiedenleut
107	after fissioning	Schmiedenleut	86	after fissioning	Schmiedenleut
76	after fissioning	Schmiedenleut	50	after fissioning	Schmiedenleut
93	after fissioning	Schmiedenleut	54	after fissioning	Schmiedenleut
95	after fissioning	Schmiedenleut	50	after fissioning	Schmiedenleut
73	after fissioning	Schmiedenleut	88	after fissioning	Schmiedenleut
69	after fissioning	Schmiedenleut	56	after fissioning	Schmiedenleut
107	after fissioning	Schmiedenleut	86	after fissioning	Schmiedenleut
81	after fissioning	Schmiedenleut	77	after fissioning	Schmiedenleut
81	after fissioning	Schmiedenleut	86	after fissioning	Schmiedenleut
52	after fissioning	Schmiedenleut	76	after fissioning	Schmiedenleut
123	after fissioning	Schmiedenleut	81	after fissioning	Schmiedenleut
89	after fissioning	Schmiedenleut	64	after fissioning	Schmiedenleut
101	after fissioning	Schmiedenleut			
68	after fissioning	Schmiedenleut			
117	after fissioning	Schmiedenleut			

**Figure 2:**
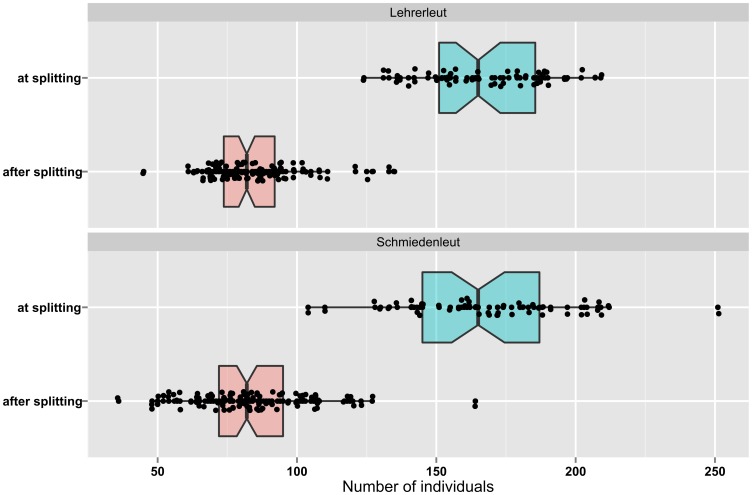
Size of the Hutterite colonies (belonging to two different endogamous subsets) at and after fissioning. Notched boxplots show the distribution of the size values (in terms of number of individuals), which are also represented by jittered dots. Overlapping of notches indicates a not significant difference at about 95% confidence [102]. Data derived from Olsen [63].

For the purposes of this study, the data for the two groups have been lumped together since the size distribution of the Lehrerleut colonies at fissioning does not significantly differ from that of the Schmiedenleut colonies. The same holds true for the size distribution of the colonies after fissioning (Fig. 2). The overall sample size is equal to 297 observations.

**Figure 3:**
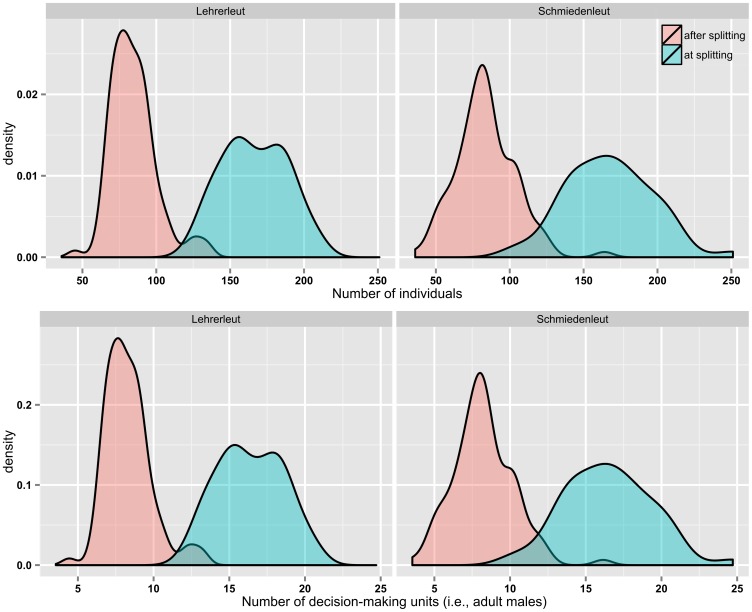
Distribution of the size of the Hutterites colonies at and after fissioning, expressed in terms of both sheer population size and decision-making units. Decision-making units correspond to baptized married males. The latter figures are calculated dividing the number of individuals at each colony (after Olsen) by the average Hutterite family size (8.16) as derived from the data by Janzen and Stanton [66].

It has been noted that the fissioning event among Hutterites can be conceived as an example of limits to group size due to cognitive constrains. It has been also noted that the Hutterite case allows linking the Johnson’s model of scalar stress, which is framed in terms of decision-making units, to the Dunbar’s model of cognitive limits to human network sizes, which is framed in terms of sheer population size. This link seems empirically supported by the following evidence, which requires preliminarily, yet concisely, summarizing the Hutterites’ decision-making organization. This is based on baptized married males and is arranged into nested levels, remarkably resembling those of the Johnson’s model previously described. A congregation of all baptized married men votes on major colony policies and selects the members of a council, which in turn is made up of five to seven men selected to serve in an executive capacity [64], [95] and whose decisions are brought before the congregation for approval [65]. If we measure the size of Hutterites colonies in terms of basal decision-making units (i.e., adult married males) rather than in terms of sheer population size, it turns out that the number of adult males at fissioning is well above the Johnson’s threshold (Lehrerleut: mean 16.4, SD 2.2; Schmiedenleut: mean 16.5, SD 2.9), while is remarkably close to it when no fission is needed (Lehrerleut: mean 8.3, SD 1.5; Schmiedenleut: mean 8.2, SD 2.0) (Fig. 3). It must be noted that the link between Johnson’s and Dunbar’s models is particularly important in archaeological perspective since, while groups’ decision-making units can be difficult to identify on material bases alone, the sheer population size (or its order of magnitude) could be relatively easier to estimate, although some inherent difficulties (to which reference has been made earlier) should be borne in mind. Before proceeding, it must be stressed that the link discussed above is not meant to suggest that Hutterites’ decision-making organization must be representative of all other human groups and cultures or, by the same token, that that specific link between population size and the number of decision-making units must be universal. Rather, on the one hand, the previous preliminary analysis is aimed at linking by means of an empirical evidence Johnson’s and Dunbar’s models, which are framed, as already stressed, in two different terms (i.e., decision-making units and sheer population number respectively). On the other hand, once the aforementioned link between the two theories has been empirically assessed, it will be the sheer number of individuals that will matter for the sake of the model proposed by this study, not the number of decision-making units.

In the logistic regression model, the community size has been entered as the independent variable *x*. On the grounds of the theoretical framework previously sketched, the absence or presence of fissioning events for each Hutterite colony has been considered expression of the absence or presence of a critical (i.e., unmanageable) level of scalar stress, that is of that *increased tension and decreased social control* [63] and of those *disruptive* and *antagonistic forces* that *increase with increasing group size* [83]. Therefore, a nominal variable with two levels, i.e. not critical/critical scalar stress, has been entered in the model as the dependent variable *y*.

Before proceeding, it has to be made clear why using logistic regression instead of simply hypothesizing the existence of scalar stress on the basis of a theoretical threshold derived from Durbar’s studies. Provided the fact that the use of Dunbar’s figures to devise a workable threshold would be complicated since a point estimate (150) as well as a range (100–230) is available, in my opinion what calls for the technique used in this study is the need to derive the probability that a group experienced a critical level of scalar stress rather than simply working out a “yes/no” response of the type one would get using a simple threshold as baseline for judgment. Instead, Olsen’s Hutterites dataset and logistic regression allow working out a model that can provide a continuum scale of probability that can be more flexible than straightjacketing the whole process into a binary response. Since elaborating further on this issue entails delving into the core of this study’s results, I prefer to provide some more comments later on.

## Results and Discussion

**Figure 4:**
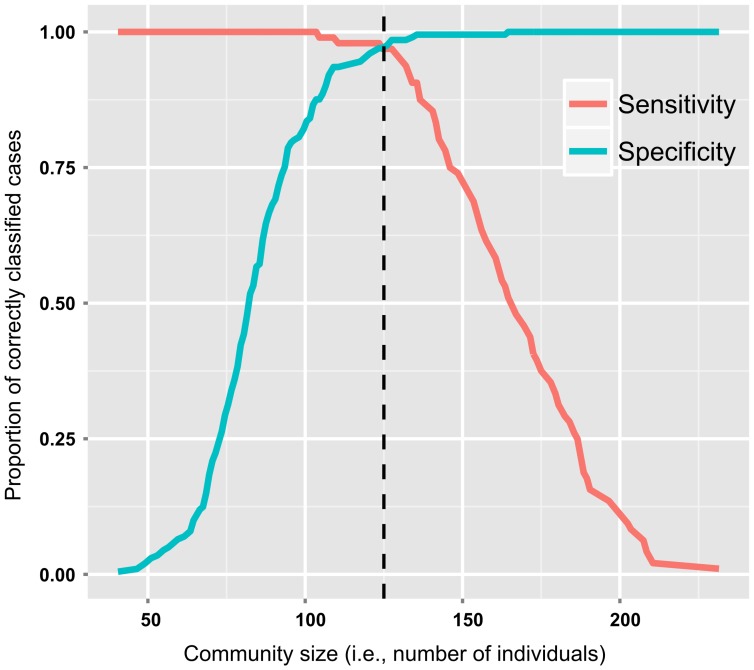
Optimal cutoff on Logistic Regression probabilities. Plot of sensitivity (percentage of correctly classified cases of critical scalar stress) and specificity (percentage of correctly classified cases of not critical scalar stress) versus community size. Reference line: intersection point at which there is a balance between sensitivity and specificity; it corresponds to the optimal cutoff on logistic regression probabilities (community size 127 = *p* 0.50). See also Table 2.

Logistic regression indicates that there is a significant relationship between the absence/presence of critical scalar stress and community size. The model adequately fits the data, as the goodness-of-fit test (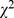 = 132.6, *df* = 124, *p* = 0.28), the percentage of concordant pairs (99.5) and the Somers’ D measure of association (0.99) indicate. The intercept (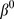) and slope (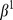), both statistically significant, are equal to −18.636 (Z = −5.96, *p*<0.001; standard error = 3.127, 95% conf. int. =  −24.764, −12.507) and 0.147 (Z = 5.98, *p*<0.001; standard error = 0.025, 95% conf. int. = 0.098, 0.196) respectively, and can be plugged into the regression model equation previously reported in order to get the probability value for any community size at hand (a script to accomplish this in the free R statistical environment [96] is available from this author upon request or from https://independent.academia.edu/GianmarcoAlberti). The model has a very good classificatory power (at 0.5 cutoff value), as reported in Table 2, which shows the percentages of correctly classified cases. The overall percentage is equal to 98%. That cutoff value is the optimal one for future classifications since it corresponds to the point that yields an approximately equal proportion between sensitivity (i.e., percentage of correctly classified cases of critical scalar stress) and specificity (i.e., percentage of correctly classified cases of not critical scalar stress) [60] (Fig. 4).

**Table 2:** Classification table reporting the overall percentage of correctly classified cases (0.5 cutoff value), and showing the very good classificatory power of the Logistic Regression model (see also Fig. 4).

from/to	not critical scalar stress	critical scalar stress	Total	% correct
**not critical scalar stress**	129	2	131	98.5% (specificity)
**critical scalar stress**	2	64	66	96.9% (sensibility)
**Total**	131	66	197	97.9%

**Figure 5:**
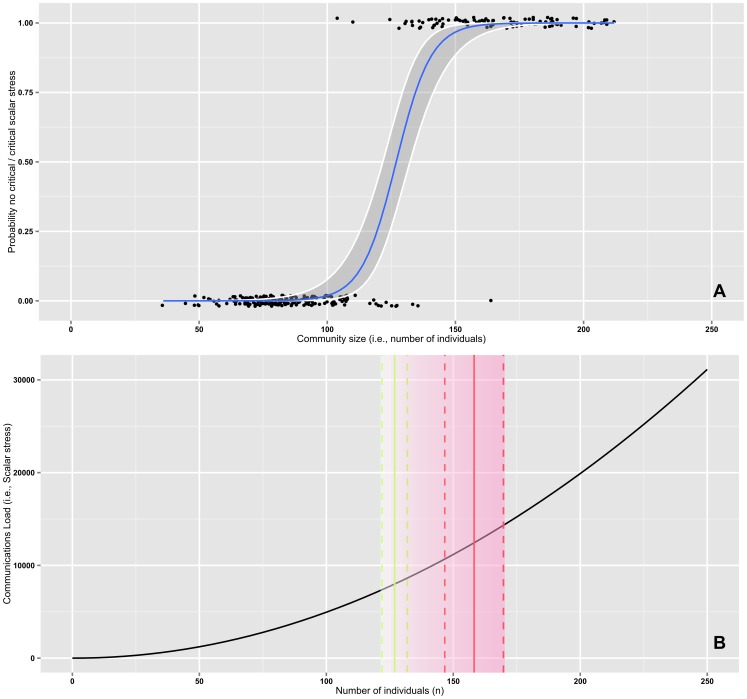
Logistic Regression model. **A**) Best fitting logistic curve (plus 95% confidence band) for the critical level of scalar stress as derived from Olsen’s Hutterite fissioning data. Vertical axis: probability of experiencing not critical/critical scalar stress; horizontal axis: community size in terms of sheer number of individuals. Lower probabilities of experiencing a critical level of scalar stress are associated with smaller community sizes and higher probabilities with larger community sizes. **B**) Scalar stress (*sensu* Johnson) chart plus critical threshold as derived by the logistic regression model. Black line: scalar stress as function of number of individuals; it is equal to (n^2^−n)/2, where n is the number of interacting subjects. Vertical lines: in green, group size threshold at which the probability of critical scalar stress begins to change from low to high (i.e., *p* = 0.50; mid-point: 126.9; 95% confidence interval: 121.9–131.9); in red, group size threshold at which the probability of critical scalar stress reaches its maximum (i.e., *p* = 0.99; mid-point: 158.2; 95% confidence interval: 146.6–169.8). See also Table 3. The gradient in the shaded area is meant to visually represent the increase in probability between the two thresholds.

As apparent from the logistic regression plot (Fig. 5A), the model indicates that lower probabilities of experiencing a critical level of scalar stress are associated with smaller group sizes and higher probabilities with larger group sizes. The analysis allows pinpointing the size above which scalar stress can be considered critical: the point where the probability begins to change from low to high (i.e., *p* = 0.50) is at size 126.9 (95% Confidence Interval: 121.9–131.9) (Fig. 5B). The maximum probability (i.e., *p* = 0.99) of critical scalar stress is reached at size 158.2 (95% CI: 146.6–169.8) as apparent from Table 3, which also shows the 95% confidence intervals for group size at increasing probability levels of critical scalar stress.

**Table 3:** Mid-point and 95% confidence limits for community size at increasing probability levels of critical scalar stress, as estimated by the Logistic Regression model.

Probability of critical scalar stress	n. of individ. (i.e., community size)	95% Lower Conf. Limit	95% Upper Conf. Limit
0.10	111.9	105.1	118.82
0.30	121.1	115.8	126.4
0.50	126.9	121.9	131.9
0.70	132.7	127.3	138.1
0.90	141.9	134.8	148.9
0.99	158.2	146.6	169.8

The predictive power of the model turns out to be very good when it is put to work on a sample of archaeological and ethnographic cases, as what follows indicates. It is expected that evidences of fissioning events or of use of integrative facilities occur in those cases for which the logistic regression model predicts a high probability of a critical level of scalar stress on the basis of population size. For the sake of model testing, the population size of the following archaeological settlements is taken into account: a) Chiamamaya, Cerro Choncaya and Sonaji, dating to the Formative Period of Bolivia’s Titicaca Basin (about 1000-800 BC), which experienced fissioning processes during the local Chiripa phase [3]; b) Broken K pueblo in US (about 1150–1280 AD) [53], which is featured by the presence of structures (i.e., kivas) with socially integrative functions [29], [97]; c) Middle Bronze Age Lipari (northeastern Sicily, Italy; about 1460-1250 BC) that is featured by the presence of an oversized structure (hut Gamma 12) likely to have been used as integrative facility, as a host of evidences (i.e., artefacts inventory, faunal data, size, and layout) would suggest [38] (see the aforementioned Fig. 1); d) Neolithic Jiangzhai (China; about 5000-4000 BC), where five oversized integrative facilities were built during the middle stage of the village development [24]; e) sixteenth-century King site in northern Georgia (US), where an oversized structure is likely to have been used as meeting house for households’ representatives in the context of decision-making [57]. For the same purpose, I also take into account the ethnographic sample collected by Adler and Wilshusen [30], [34], consisting of villages whose population size has been estimated and where integrative facilities are documented.

Table 4 reports the probability and 95% confidence limits (which can be calculated by the aforementioned R script) of experiencing critical scalar stress as predicted by the logistic regression model. It is apparent that the fissioning events located by Bandy in the Titicaca’s Basin villages are coupled with the highest probability figures. The same holds true for Broken K pueblo and the King site. As for Lipari, the village size estimate allows predicting between 0.88 and 0.99 probability of experiencing critical scalar stress. The cases of Titicaca’s Basin and Jiangzhai turn out to be particularly intriguing because they offer the opportunity to test if the absence of integrative facilities was coupled with a low level of critical scalar stress, i.e. with a population size below the critical level pinpointed by the proposed model. It is apparent that the Jiangzhai’s early and late phases have the lowest probability of critical scalar stress, while the highest probability is predicted for the middle phase. Remarkably, the construction and use of the integrative facilities trace back to the very phase (namely, the middle) for which the highest probability of critical scalar stress is predicted. Both the preceding and subsequent phase, featured by lower population figures, did lack integrative structures. Incidentally, this provides support to Lee’s [24] hypothesis about the correlation between high population density and the construction of large non-domestic buildings during that particular phase. The Jiangzhai case is remarkably consistent with the Titicaca’s Basin scenario as portrayed by Bandy. Not only, as stressed above, the logistic regression model returns the highest probability of scalar stress for the Titicaca’s sites considered, but, as Bandy stresses, fissioning events (expression of critical level of scalar stress) in his study area were coupled with a lack of integrative structures and mechanisms. Remarkably, when the latter were created, they put an end to the fissioning processes or, as Bandy puts it, *had the effect to obviating the need of fissioning* [3]. While a similar cross-check of the model would be desirable also on ethnographical grounds (and could be object of future works), I believe that the Chinese and Titicaca cases, as diverse as they are in chronological and cultural settings, could represent a promising, yet small, supporting evidence of the goodness of the proposed model.

**Table 4:** Probability and 95% confidence limits of experiencing critical scalar stress for a sample of archaeological (1–9) and ethnographic cases (10–29), as predicted by the Logistic Regression model.

	archaeological/ethnographic cases	n. of indiv.(i.e., community size)	Probability of criticalscalar stress	95% LowerConf. Limit	95% UpperConf. Limit
1	Chiaramaya	186	1.00	0.99	1.00
2	Cerro Choncaya	157	0.99	0.94	1.00
3	Sonaji	277	1.00	1.00	1.00
4	Broken K	182	1.00	0.99	1.00
5	Lipari	150	0.97	0.88	0.99
6	Jiangzhai I early	105	0.04	0.01	0.12
7	Jiangzhai I middle	255	1.00	1.00	1.00
8	Jiangzhai I late	71	0.00	0.00	0.00
9	King	235	1.00	1.00	1.00
10	Arapesh	250	1.00	1.00	1.00
11	Baktman	250	1.00	1.00	1.00
12	Bororo	200	1.00	1.00	1.00
13	Dogon	210	1.00	1.00	1.00
14	Elema	500	1.00	1.00	1.00
15	Etoro	75	0.00	0.00	0.01
16	Fang	250	1.00	1.00	1.00
17	Great Basin	75	0.00	0.00	0.01
18	Kiwai	250	1.00	1.00	1.00
19	Maidu	175	1.00	0.99	1.00
20	Mandan	320	1.00	1.00	1.00
21	Mundurucu	200	1.00	1.00	1.00
22	N. Pomo	150	0.97	0.89	0.99
23	New Ireland	220	1.00	1.00	1.00
24	Orokaiva	150	0.97	0.88	0.99
25	Tareumiut	100	0.02	0.00	0.08
26	Tipirape	200	1.00	1.00	1.00
27	Wogeo	100	0.02	0.00	0.08
28	Yuman	150	0.97	0.89	0.99
29	Yurok	200	1.00	1.00	1.00

The results of the analysis of the ethnographic sample quite closely mirror those deriving from the archaeological cases. It is apparent that structures for social integration feature those villages having the highest probability of experiencing critical scalar stress. The few exceptions (Table 4, nos 15, 17, 25, 27) can be accounted for by problems in deriving exact estimates for some community sizes, leading to conservative figures [34]. It is worthy of note that the communities to which reference has been made in the introduction of this study (namely, Yanomama and Conambo), and for which scholars have stressed the existence of conflicts and tensions, leading to either fissioning events [10] or practices of social integration [11], [98], have size well past the threshold located by the logistic regression model.

As for the latter aspect, namely the size of the cases (both archaeological and ethnographical) on which the model has been put to work, it must be noted that while a certain degree of variability in community size exists, and while extreme cases are documented, a number of instances are nonetheless close to the threshold derived by logistic regression (i.e., 127). By inspecting Table 4 (nos 1, 2, 4, 5, 6, 18, 21, 24, 27), it is possible to see that a number of community sizes approach logistic regression’s critical threshold both from below and above (e.g., 100, 105, 150, 182). While a discussion of the scenarios that open up at various community sizes (e.g., 105 vs. 120 vs. 127 vs. 155), in terms of the probability returned by the logistic regression, is provided in the next section of this paragraph, to understand the progression of the probability across the critical thresholds it can be useful to keep in mind that 127 is the community size at which the probability of critical scalar stress begins to change from low to high, i.e. where *p* = 0.50. As community size becomes progressively smaller than 127, *p* progressively decreases as well. By the same token, as the former increases, the latter progressively increases above 0.50, until it saturates (i.e., it reaches 0.99) at community size 158.

To comment further, yet concisely, on the issue previously touched upon (namely, why using logistic regression instead of a simple threshold derived from Dunbar’s findings), it should be apparent how probability figures prove to be a more flexible means to assess the degree to which a group was likely to experience critical scalar stress. For instance, by using as baseline the Dunbar’s range to which reference has been made earlier in this work, a community of size 120 could be thought of as experiencing a critical level of scalar stress since its size is past the lower hinge of the Dunbar’s threshold. Yet, the proposed model allows estimating a low probability (*p* = 0.27; 95% CI: 0.14–0.45). As a case in point, see for example the earliest phase of Jiangzhai. Without logistic regression, its population size (105) would be thought of as generating a critical level of scalar stress, whereas the model indicates that the opposite is true (*p* = 0.04; 95% CI: 0.01–0.13). By the same token, suppose we have two groups of size 127 and 155 respectively, for which (using the simple aforementioned threshold) we would conclude that both experienced scalar stress. Instead, the logistic regression model allows indicating a more nuanced scenario, indicating that the first is about the threshold of critical scalar stress (*p* = 0.50; 95% CI: 0.33–0.68) while the second has a higher probability (*p* = 0.98; 95% CI: 0.93–0.99). These examples should underscore the utility of the logistic regression approach versus the use of a theoretically derived threshold.

Moving toward the end of this study, one may wonder why the model here proposed, based on the Hutterites’ data, should have a more general validity, i.e. being cross-culturally valid. I believe it is because the cognitive constrains to groups’ size highlighted by Jonson and Dunbar, even from two slightly different perspectives, are inherent to humans. In this respect, and more importantly, a number of studies have tested the implications of Dunbar’s findings for human groups on the grounds of a body of data encompassing not only hunter-gatherers and small-scale horticulturalists, but also Neolithic groups, ancient and modern military organizations, modern agricultural groups (among which the Hutterites), modern corporations and business organizations [59], [68], [88], [99], up to modern on-line social networks [87], pointing that functional limits to groups size are likely to apply universally [59], [88]. The cases-study discussed in this work, spanning a variety of archaeological and ethnographic contexts, from New to Old World, from Neolithic China to pre-contact America, are consistent with that scenario, would point to a broader validity of the theory of cognitive constrains to groups size, and may hopefully come as a significant addition to the existing case studies.

Overall, it is apparent that, by its ability to devise probability figures, the model proposed can allow expecting (or not) the presence of evidences of integrative mechanisms when group size is known beforehand, so providing the possibility to tailor the researches on field. On the other hand, when group size is known and there is evidence of mechanisms liable to be interpreted as socially integrative, the model allows evaluating (in terms of probability) whether or not critical levels of scalar stress could have been present and could possibly account for the integrative nature of such evidences, or if other explanations are needed instead. In a sense, the model ultimately provides grounds to assess the probability that a group reached a *hot spot* [26] of size development that was critical for its internal cohesion, and which would had required those *formal methods of minimizing stress and maximizing interaction* [100] to which references have been made earlier in this study (i.e., fissioning, places and mechanisms for social integration, rituals, stylistic displays, shared food consumption) and that are all amenable to further archaeological investigations.

## Conclusions

This study has attempted to devise a predictive model of scalar stress on the basis of population size, drawing upon Johnson’s theory of scale-related social issues and on Dunbar’s theory of cognitive constrains to human groups size. The latter has enabled to re-express Johnson’s findings, which are couched in terms of decision-making units, in terms of sheer population size instead.

A logistic regression model has been build relying upon Olsen’s data on the Hutterites’ communities size at and after fissioning, which have been considered expression of critical level of scalar stress in the light of the theoretical framework sketched at the beginning of this work. The model, on the one hand, has confirmed the existence of a significant relationship between critical scalar stress and group size, setting the issue in firmer numerical and statistical grounds. Further, the analysis allowed pinpointing a critical scalar stress threshold at size 127, with 95% confidence interval between 122 and 132. This threshold represents the size at which the probability of experiencing critical scalar stress changes from low to high (i.e., *p* = 0.50); the latter attains its maximum (i.e., *p* = 0.99) at population size 158 (95% confidence interval: 147–170). When experiencing critical level of scalar stress, the community organization must change. A number of human responses are available, such as splitting, decision-making reshaping, cohesion-building practices (e.g., food consumption, stylistic display), use of integrative facilities, or even leadership development, which are all contingent and context-specific and which have been reviewed in the first part of this study.

Notably, the model proved good when tested against a sample of archaeological and ethnographic cases, overall indicating (and formally confirming) that fissioning events and integrative facilities are coupled with high probabilities of critical level of scalar stress. On the other hand, and more importantly from a practical standpoint, the analysis allowed deriving the intercept and slope of the logistic regression model, which can be plugged into the regression equation by anyone who is interested in estimating, for the sake of any further archaeological/anthropological explanation, the probability for a community of experiencing critical level of scalar stress. It must be acknowledged that two achievements of the present study could be amenable to future investigations, as stressed by the reviewers of this work. While, earlier in this study, promising evidence has been discussed pointing to a connection between low levels of scalar stress and the absence of integrative facilities, it would be interesting to broaden the ethnographical and archaeological data in order to provide additional supporting evidences. In particular, emphasis could be placed on multiphase archaeological settlements in a way similar to what has been done in this study in relation to the evidence from the Jiangzhai village. Finally, another future avenue of inquiry could be to feed additional datasets into logistic regression in order to check whether the intercept and slope change significantly across them. This would be an interesting addition to the present study and could be accomplished provided that rich (i.e., of adequate size) and detailed datasets, similar to the Olsen’s one used in this study, could be available or located in literature.
